# Surface Modification of SPIONs in PHBV Microspheres for Biomedical Applications

**DOI:** 10.1038/s41598-018-25243-9

**Published:** 2018-05-08

**Authors:** Maizlinda I. Idris, Jan Zaloga, Rainer Detsch, Judith A. Roether, Harald Unterweger, Christoph Alexiou, Aldo R. Boccaccini

**Affiliations:** 1 Institute of Biomaterials, Department of Materials Science and Engineering, University of Erlangen-Nuremberg, Cauerstrasse 6, 91058 Erlangen, Germany; 2 Department of Materials Engineering and Design, Faculty of Mechanical and Manufacturing, Universiti Tun Hussein Onn Malaysia, 86400 Parit Raja, Batu Pahat, Johor Malaysia; 3 Department of Otorhinolaryngology, Head and Neck Surgery, Section of Experimental Oncology and Nanomedicine (SEON), Else Krӧner-Fresenius-Stiftung Professorship, University Hospital Erlangen, Glückstrasse 10a, 91054 Erlangen, Germany; 4 Institute of Polymer Materials, Department of Materials Science and Engineering, University of Erlangen-Nuremberg, Martensstrasse 7, 91058 Erlangen, Germany

## Abstract

Surface modification of superparamagnetic iron oxide nanoparticles (SPIONs) has been introduced with lauric acid and oleic acid via co-precipitation and thermal decomposition methods, respectively. This modification is required to increase the stability of SPIONs when incorporated in hydrophobic, biodegradable and biocompatible polymers such as poly (3-hydroxybutyrate-co-3-hydroxyvalerate) (PHBV). In this work, the solid-in-oil-in-water (S/O/W) emulsion-solvent extraction/evaporation method was utilized to fabricate magnetic polymer microspheres incorporating SPIONs in PHBV. The prepared magnetic PHBV microspheres exhibited particle sizes <1 µm. The presence of functional groups of lauric acid, oleic acid and iron oxide in the PHBV microspheres was confirmed by Fourier Transform Infrared spectroscopy (FTIR). X-ray diffraction (XRD) analysis was performed to further confirm the success of the combination of modified SPIONs and PHBV. Thermogravimetric analysis (TGA) indicated that PHBV microspheres were incorporated with SPIONs^Lauric^ as compared with SPIONs^Oleic^. This was also proven via magnetic susceptibility measurement as a higher value of this magnetic property was detected for PHBV/SPIONs^Lauric^ microspheres. It was revealed that the magnetic PHBV microspheres were non-toxic when assessed with mouse embryotic fibroblast cells (MEF) at different concentrations of microspheres. These results confirmed that the fabricated magnetic PHBV microspheres are potential candidates for use in biomedical applications.

## Introduction

Poly (3-hydroxybutyrate-co-3-hydroxyvalerate) (PHBV) is a biodegradable and biocompatible biopolymer derived from several types of bacteria such as *Ralstonia eutropha*^1–3^. These properties make PHBV suitable for biomedical applications such as scaffolds for bone and soft tissue engineering^4–6^, fibrous mats for wound healing^7^ and microspheres for drug delivery applications^8–10^. Recently, synthetic polymer microspheres such as polyethyleneimine^11^ and poly(lactide-co-glycolide)^12^ have been incorporated with iron oxide nanoparticles for targeted drug delivery and magnetic resonance imaging (MRI) monitoring, respectively. However, synthetic based-polymers have some drawbacks related to the polymer structure and charges from polyethyleneimine that can cause toxicity to cells^13^. As an alternative new system, Li *et al*.^14^ successfully prepared multifunctional PHBV microspheres incorporating SPIONs. They reported that these magnetic biopolymer microspheres could exhibit significant effect on magnetic contrast capability suitable for MRI application, demonstrating that the microspheres could be suitable also as drug delivery vehicles, in addition, such PHBV/SPION microspheres exhibited biocompatibility towards human T-lymphoma suspension cells and adherent colon carcinoma HT-29 cells. On the other hand, the application of bare SPIONs has shown some limitations as the magnetic properties diminish due to exposure to air^15^ or due to variations in storage conditions^16^. Therefore, it is very important to introduce surface modifications or protective coating on SPIONs to provide protection to their intrinsic properties while maintaining their stability. The first section of this study involves the modification of SPIONs to obtain adequate stability of SPIONs in suspension and suitable interaction between hydrophobic PHBV solution and the surfaces of SPIONs during preparation of magnetic microspheres, which is presented in the second part of the study. The surface of the SPIONs was modified with two types of fatty acid, namely lauric acid and oleic acid, through co-precipitation and thermal decomposition methods. It is understood that the co-precipitation method produces particles with a wide particle size distribution, as compared with the thermal decomposition method which generates a narrow particle size distribution^15^. In this study, the production of PHBV microspheres with modified SPIONs was chosen via emulsion-solvent extraction/evaporation method. This method has been described in detail by Li and co-authors^14^. A further goal of this investigation is thus to provide findings related to the chemical, structural, thermal and magnetic properties as well as *in vitro* biocompatibility of the prepared magnetic PHBV microspheres combined with SPIONs modified with lauric acid and oleic acid. As far as the authors know, these two types of modifications on SPIONs for their interaction with PHBV have not been investigated and compared before.

## Results and Discussion

### Microstructure, chemical and physical properties of microspheres

**Figure 1:**
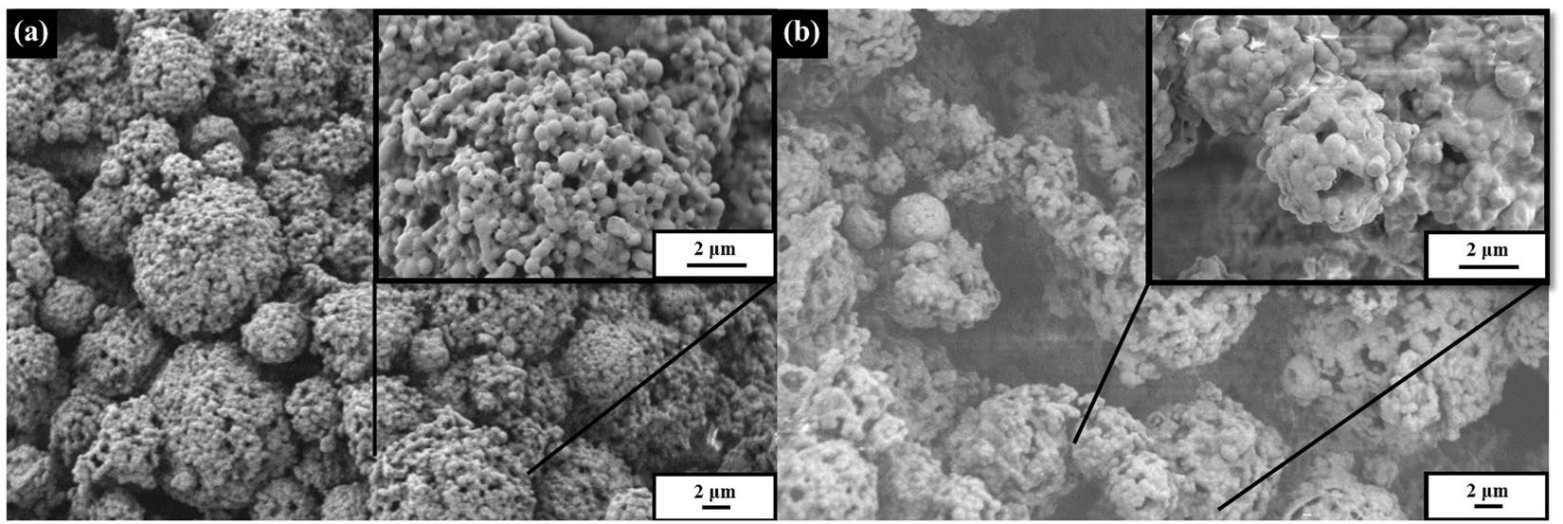
Surface morphology of (**a**) PHBV and (**b**) PHBV containing SPIONs^Oleic^.

The surface morphology of PHBV microspheres and PHBV microspheres incorporated with SPIONs^Oleic^ was evaluated by scanning electron microscopy (SEM) (Fig. 1). It can be observed that the particle size of PHBV and PHBV/SPIONs microspheres was <1 μm and they had a tendency to agglomerate. It was reported by Chang and Niklason^17^ that the size of blood vessels can be divided into three categories which are (i) microvessels (<1 mm), (ii) small vessels (1–6 mm), and (iii) large vessels (>6 mm in diameter). Therefore, it can be seen that the agglomeration of PHBV or PHBV/SPIONs microspheres will not contribute to the blockage of blood vessels when utililsed as local drug delivery device, as intended. By visual inspection, it was seen that PHBV microspheres are white and tend to change into brownish colour after incorporation with SPIONs. This indicates that while the larger amount of SPIONs was incorporated in PHBV, a low amount of SPIONs might have also attached on the surface of the microspheres during the fabrication process.

**Figure 2:**
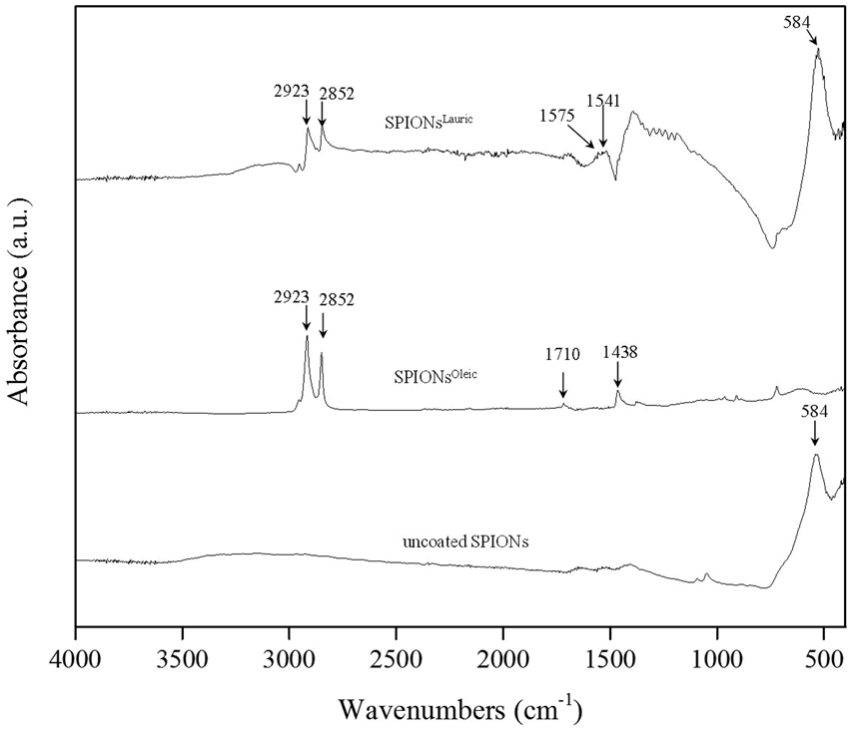
FTIR of uncoated SPIONs, SPIONs^Oleic^ and SPIONs^Lauric^. The different relevant peaks are discussed in the text.

**Figure 3:**
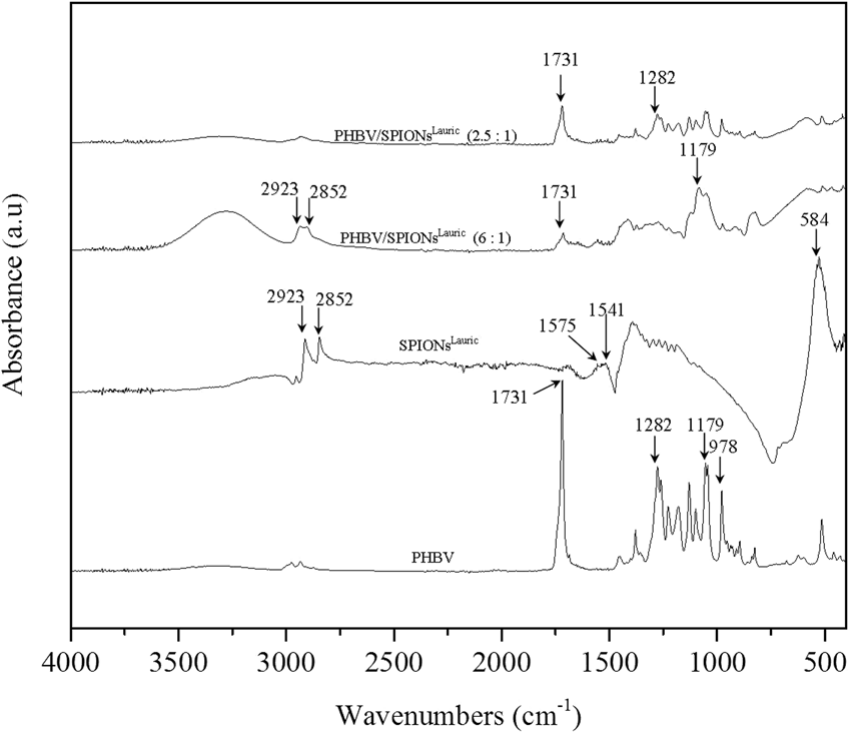
FTIR results on PHBV, SPIONs^Lauric^ and PHBV incorporating SPIONs^Lauric^. The relevant peaks are discussed in the text.

**Figure 4:**
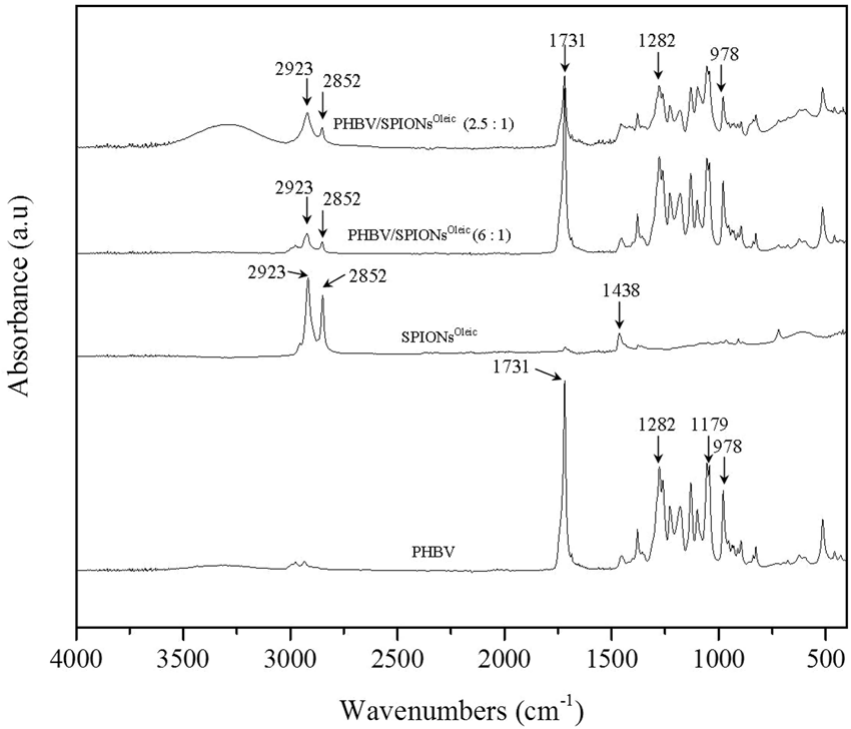
FTIR results on  PHBV, SPIONs^Oleic^ and PHBV containing SPIONs^Oleic^. The characteristic peaks are discussed in the text.

FTIR spectra performed for uncoated SPIONs, SPIONs^Oleic^ and SPIONs^Lauric^ are shown in Fig. 2. The IR absorption band in the range of 630–550 cm^−1^ is attributed to the vibration of Fe-O from Fe_3_O_4_ which can be seen from both uncoated and coated SPIONs^18,19^. Then, absorption bands at 2852 cm^−1^ and 2923 cm^−1^ are assigned to the symmetric and asymmetric C–H stretching from oleic and lauric acids^20^. The characteristic band at 1710 cm^−1^ is related to C=O stretching of the carboxylic group from oleic acid^21,22^. Further, the oleate ion trapped on the magnetite surface can result in the symmetric vibration of COO^−^ detected at 1438 cm^−1 ^^23^. SPIONs^Lauric^ showed broad bands at 1541 cm^−1^ and 1575 cm^−1^ which are representative of the carboxylate group^16^. Figures 3 and 4 depict the PHBV infrared spectrum with a sharp absorption band at 1731 cm^−1^, which indicates the stretching mode of C=O in the crystalline phase of PHBV. The absorption bands at 1282 cm^−1^ and 1179 cm^−1^ can be ascribed to the crystalline and amorphous parts via stretching modes of C–O–C^24,25^. Also, the typical bands from 800 to 975 cm^−1^ correspond to symmetric –C–O–C– stretching vibration as well^26^. These FTIR analyses also confirmed the successful incorporation of PHBV microspheres with surface modified SPIONs.

**Figure 5:**
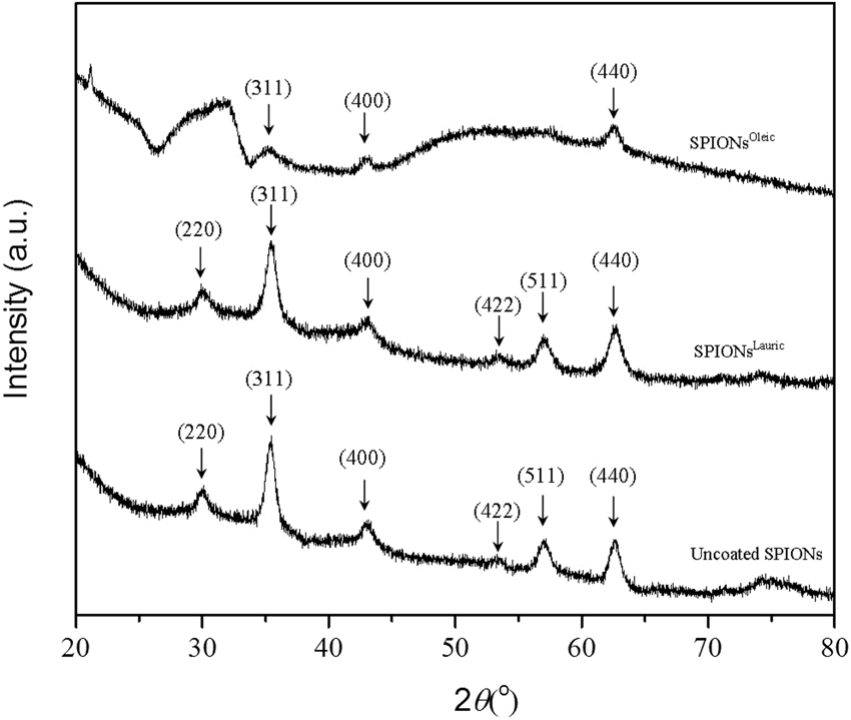
XRD patterns of uncoated SPIONs, SPIONs^Lauric^ and SPIONs^Oleic^. The characteristic peaks of Fe_3_O_4_ are marked.

**Figure 6:**
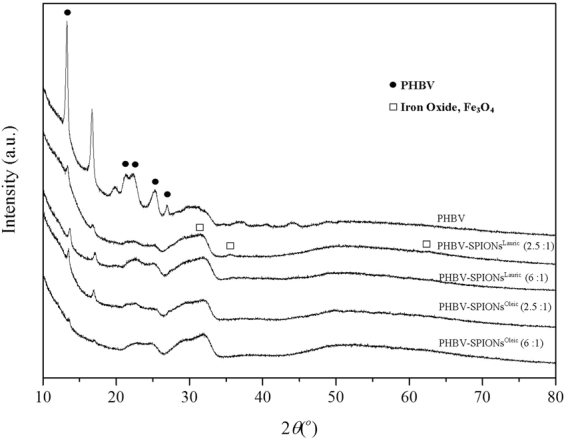
XRD patterns of PHBV microspheres, PHBV microspheres containing SPIONs^Lauric^ and SPIONs^Oleic^.

The crystalline structure of uncoated SPIONs, SPIONs^Lauric^, SPIONs^Oleic^, PHBV and magnetic PHBV microspheres was characterised via X-ray powder diffraction. Figure 5 shows the spectra of uncoated and coated SPIONs which are similar to the spectrum of magnetite (Fe_3_O_4_). Particularly, the peaks at 2θ equal to 30.1°, 35.4°, 43.1°, 53.4°, 57.1°, and 62.6° can be indexed as (220), (311), (422), (400), (511), and (440) lattice planes of cubic magnetite, respectively^27,28^. The characteristic peaks of PHBV in all samples can be identified in Fig. 6. The peaks related to PHBV are at 13.4°, 16.9°, 21.3°, 22.4°, 26° and 27°, which correspond to the (020), (110), (101), (111), (130) and (040) planes, respectively. Two strong peaks at 13.4° and 16.9° indicate the existence of orthorhombic unit cells in PHBV^29,30^.

**Figure 7:**
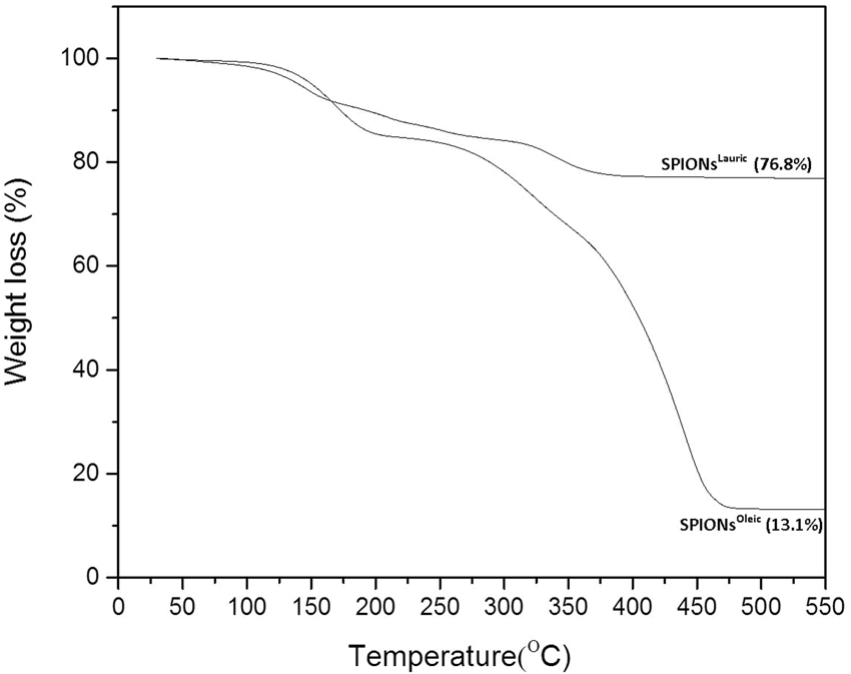
Thermogravimetric curves of SPIONs^Lauric^ and SPIONs^Oleic^ showing the percentage of weight loss (%) between 27 °C and 550 °C under nitrogen flow at the heating rate of 10 °C/min.

The thermogravimetric curves of SPIONs^Lauric^ and SPIONs^Oleic^ are shown in Fig. 7. It can be seen that SPIONs^Oleic^ were decomposed, with higher percentage weight loss of 86.9% as compared with SPIONs^Lauric^, at 550 °C. The remaining weight was attributed to iron oxide nanoparticles, which was 13.1%. This result showed that the thermal decomposition has allowed oleic acid to coat or cap with iron oxide at a lower volume. This behaviour may be due to the fact that the method was specifically designed for the synthesis of iron oxide nanoparticles in the monodispersed arrangement^31,32^. Hufschmid *et al*.^32^ obtained the thermogravimetric curve of iron (III) oleate which is similar to the present findings involving SPIONs^Oleic^. They observed that the decomposition of organic compounds between 190 °C and 330 °C is mainly due to the dissociation of the oleate ligands.

**Figure 8:**
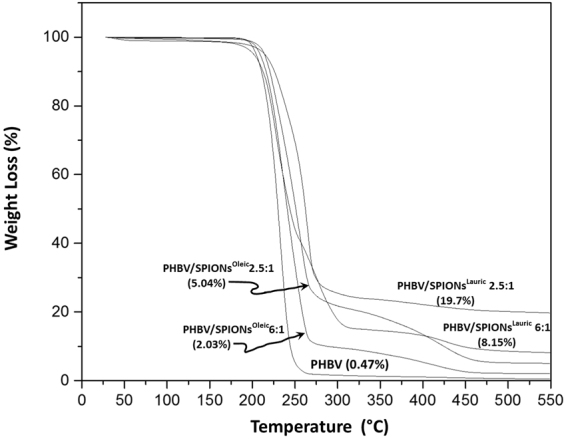
Thermogravimetric curves of PHBV, PHBV/SPIONs^Lauric^ and PHBV/SPIONs^Oleic^ showing the percentage of weight loss (%) between 27 °C and 550 °C under nitrogen flow at the heating rate of 10 °C/min.

The thermogravimetric curves of PHBV, PHBV/SPIONs^Lauric^ and PHBV/SPIONs^Oleic^ are presented in Fig. 8. It can be observed that PHBV/SPIONs^Lauric^ both at low and high mass ratios exhibit higher percentage loading of SPIONs, as compared to PHBV/SPIONs^Oleic^. This condition can be related to the use of DCM as an organic solvent to dissolve the PHBV during the production of magnetic PHBV microspheres. It has been reported that the colloidal stability of oleic acid can be influenced by the dielectric constant of the organic solvent. This result was reported by López-López *et al*.^33^ showing that oleic acid cannot be dispersed in organic solvents that have a dielectric constant higher than 5 while DCM shows a value of 8.5. The polarity of DCM should cause a gradual collapse of oleic acid tails, thus increasing the lyophobic attraction until the thermal energy becomes insufficient to keep the suspension stable^33,34^. This effect led to a major loss of SPIONs coated with oleic acid during the preparation of the magnetic PHBV microspheres. The loading and encapsulation efficiencies of PHBV containing SPIONs^Lauric^ and SPIONs^Oleic^ at different mass ratios were also calculated and results are shown in Table 1.

**Table 1:** Loading and encapsulation efficiencies of PHBV/SPIONs^Lauric^ and PHBV/SPIONs^Oleic^ at different mass ratios.

	PHBV/SPIONs^Lauric^ (6:1)	PHBV/SPIONs^Lauric^ (2.5:1)	PHBV/SPIONs^Oleic^ (6:1)	PHBV/SPIONs^Oleic^ (2.5:1)
Loading efficiency (%)	9.5	22.9	2.9	7.2
Encapsulation efficiency (%)	66.2	80.1	20.4	25.3

The results show that the loading efficiency of PHBV/SPIONs^Lauric^ at low and high mass ratio was 9.5% and 22.9%, respectively, and the encapsulation efficiency was 66.2% and 80.1%, respectively. It reveals that the loading and encapsulation efficiencies increase as the mass or percentage of SPIONs^Lauric^ increased in PHBV. These findings agree with the previous work by Nkansah *et al*.^35^ when 50% w/w magnetite was loaded into PLGA polymer via oil in water emulsion method. They found that the encapsulation efficiencies were about 80–100%. On the other hand, PHBV containing SPIONs^Oleic^ exhibited very low loading and encapsulation efficiencies at both mass ratios. It can be seen that a similar trend was discovered by Okassa *et al*.^36^ when 100% w/w magnetite nanoparticles coated with oleic acid were encapsulated into PLGA polymer. They found that only 13.5% of magnetite nanoparticles were successfully loaded into the PLGA matrix. In addition, Liu *et al*.^37^ applied 200% w/w of oleic acid-coated magnetite nanoparticles to encapsulate them into PLGA. They revealed that the highest content of magnetite nanoparticles incorporated in the polymer matrix was 60%. It can be seen that the different types of hydrophobic surfaces of magnetic nanoparticles affect the loading and encapsulation efficiencies in synthetic polymers.

**Figure 9:**
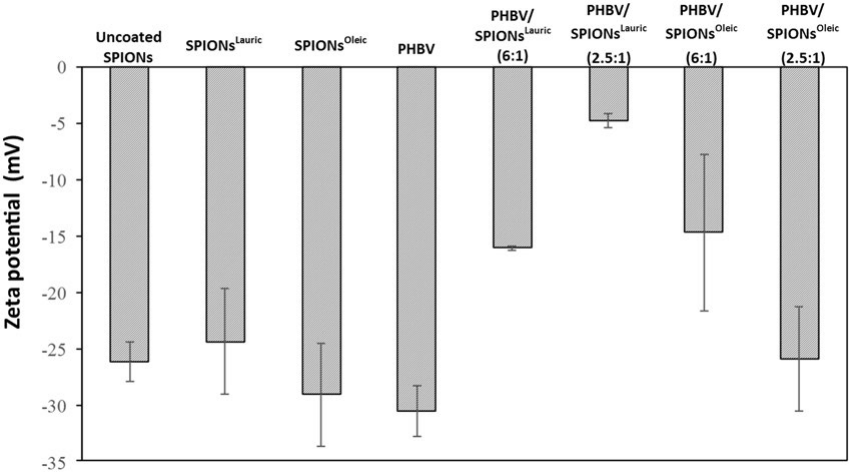
Zeta potential of uncoated SPIONs, SPIONs^Lauric^, SPIONs^Oleic^, PHBV and magnetic PHBV microspheres suspended in deionised water.

**Figure 10:**
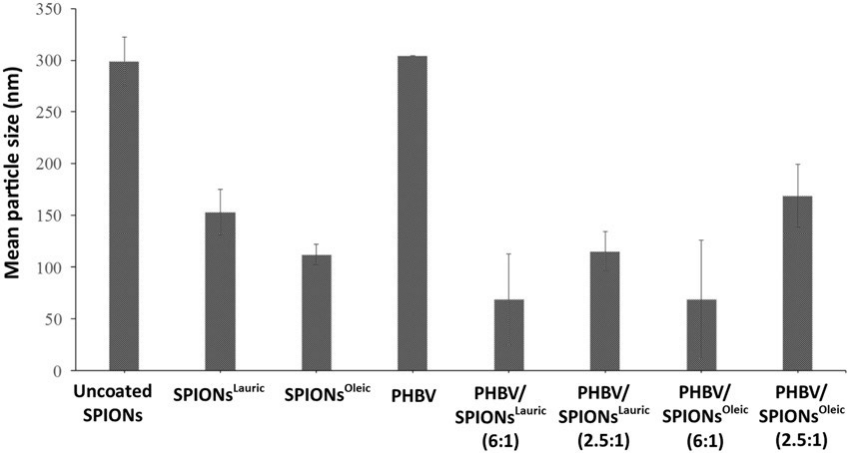
The mean particle size of uncoated SPIONs, SPIONs^Lauric^, SPIONs^Oleic^, PHBV and magnetic PHBV microspheres.

Zeta potential measurements were carried out by suspending uncoated SPIONs, SPIONs^Lauric^, SPIONs^Oleic^, PHBV and magnetic PHBV microspheres in deionised water (Fig. 9). It can be seen that the zeta potential varies from PHBV microspheres (−30.6 mV), uncoated SPIONs (−26.2 mV), SPIONs^Lauric^ (−24.4 mV) and SPIONs^Oleic^ (−29.1 mV) to magnetic PHBV microspheres (−26 mV to −4.8 mV). This behaviour is in agreement with results obtained from previous work by Li *et al*.^14^ when PHBV microspheres incorporated either with SPIONs^Lauric^ resulted in higher negative zeta potential as compared to pure PHBV microspheres. The result indicates that the dispersion and stability of magnetic PHBV are favourable in aqueous medium. Next, Fig. 10 shows the mean particle size of uncoated SPIONs, SPIONs^Lauric^, SPIONs^Oleic^, PHBV and magnetic PHBV microspheres. It shows that PHBV microspheres exhibit larger particle size (300 nm) compared to magnetic PHBV microspheres (68 nm–168 nm). In general, the particle size of magnetic PHBV microspheres was found to be smaller and this condition may be due to the production method (S/O/W) which involved homogenizer, sonicator and emulsifier; their activities at higher impact and energy possibly reduced the size of the particles. Moreover, the incorporation of SPIONs^Lauric^ and SPIONs^Oleic^ at higher loadings may have increased the instability of the emulsion which induced the formation of larger particle size as compared with the lower loading^14^.

The magnetic susceptibility was measured based on the iron content in 1 mg of the produced magnetic microspheres (Table 2). It was confirmed that the magnetic susceptibility increases as the iron content increases. It was also observed that PHBV/SPIONs^Lauric^ exhibited higher magnetic susceptibility values (per mg sample) of 4 × 10^−4^ ± 2 × 10^−6^ and 2 × 10^−3^ ± 2 × 10^−5^, at lower and higher mass ratios of SPIONs^Lauric^, respectively. This result can be related to the higher iron content in the PHBV matrix. On the other hand, the lower loading and encapsulation efficiencies of SPIONs coated with oleic acid led to the lower values of magnetic susceptibility.

**Table 2:** Magnetic susceptibility and iron content, µg mg^−1^ in PHBV/SPIONs^Lauric^ (6:1), PHBV/SPIONs^Lauric^ (2.5:1), PHBV/SPIONs^Oleic^ (6:1) and PHBV/SPIONs^Oleic^ (2.5:1).

	PHBV/SPIONs^Lauric^ (6:1)	PHBV/SPIONs^Lauric^ (2.5:1)	PHBV/SPIONs^Oleic^ (6:1)	PHBV/SPIONs^Oleic^ (2.5:1)
Magnetic susceptibility (mg^−1^ sample)	4 × 10^−4^ ± 2 × 10^−6^	2 × 10^−3^ ± 2 × 10^−5^	6 × 10^−6^ ± 1 × 10^−7^	7 × 10^−6^ ± 5 × 10^−7^
Iron content (µg mg^−1^ sample)	72.8	155.7	9.7	60

### Cell biology

**Figure 11:**
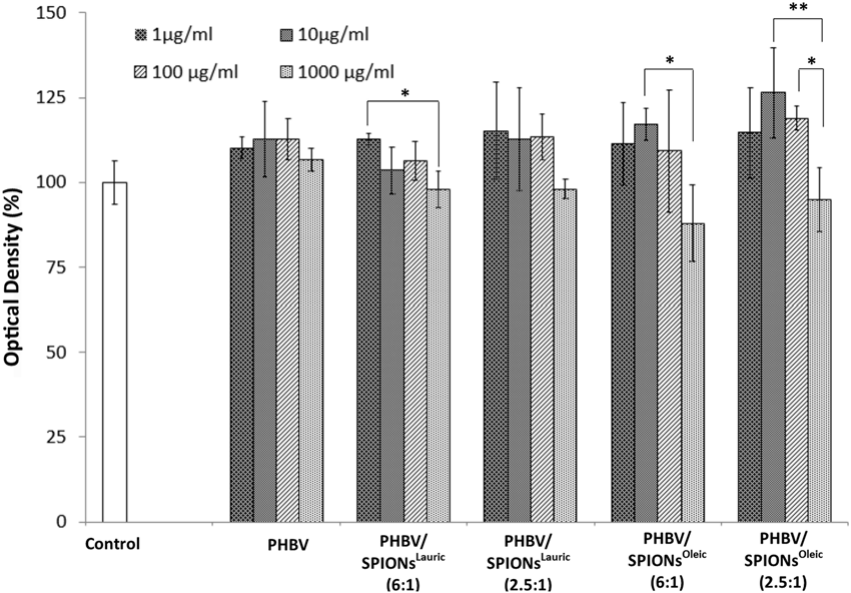
Cell viability of MEF cells in contact with extracts from PHBV-based microspheres at concentrations of 1 μg/ml, 10 μg/ml, 100 μg/ml and 1000 μg/ml after 24 hours incubation time. Asterisks denote significant difference, *p < 0.05 and **p < 0.01 (Bonferroni’s post-hoc test was used).

**Figure 12:**
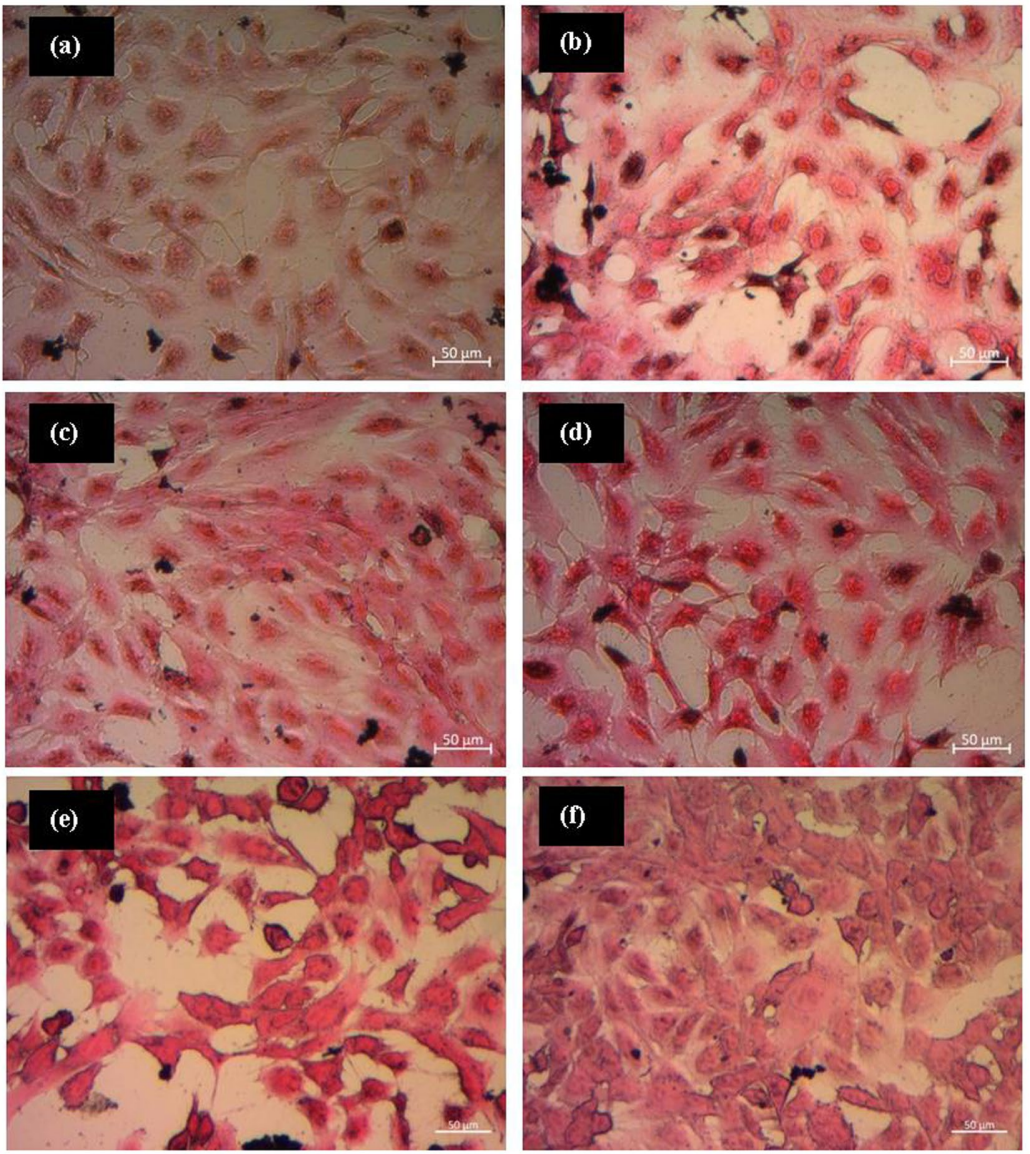
MEF cells in contact with extracts from  PHBV-based microspheres at the 1000 μg/ml concentration: (**a**) control, (**b**) pure PHBV, (**c**) PHBV/SPIONs^Lauric^ 6:1, (**d**) PHBV/SPIONs^Lauric^ 2.5:1, (**e**) PHBV/SPIONs^Oleic^ 6:1,and (**f**) PHBV/SPIONs^Oleic^ 2.5:1.

Cytotoxicity effects of pure PHBV and PHBV/SPION microspheres at different concentrations, from 1 µg/ml to 1000 µg/ml, were evaluated on MEF cells. As shown in Fig. 11, cell viability was measured using the WST-8 assay. The cell viability in all concentrations of fabricated microspheres was found to be higher as compared to the control condition. Kanemura *et al*.^38^ determined that the quantitative result of WST-8 assay indicates the number of viable cells and their proliferation activity, which are based on their metabolic activity. Also, previous researchers^39–41^ applied WST-8 cytocompatibility assay in order to investigate *in vitro* toxicity of iron oxide nanoparticles. Following this, our results show that the cell metabolic activity increases with PHBV microspheres and PHBV containing SPIONs^Lauric^ and SPIONs^Oleic^. Particularly, the stimulation effect to cell viability at a lower concentration is more pronounced, and the slightly reduction at the highest concentration does not seem to contribute to toxic effects. This condition anticipates the biocompatible behaviour of PHBV microspheres^42,43^. Also, Zhu *et al*.^44^ found that PHBV microspheres have potential as scaffold to guide and support liver cell growth. They revealed that human hepatoma cell lines (HepG2 and Hep3B) seeded on PHBV microspheres successfully secreted albumin at rates 2–4 times higher than the positive control. Recently, Solar *et al*.^45^ developed PHBV incorporated with SPIONs and the antibiotic certiofur (CEF). They performed toxicological studies on PHBV, PHBV/CEF, PHBV/SPION and PHBV/CEF/SPION at concentrations of 0.1 µg/ml up to 10 000 µg/ml with the HepG2 cell line. It was confirmed that all prepared concentration levels did not involve any significant cell death after being incubated for 24 hours. Figure 12 shows representative images of the MEF cell morphology in contact with extracts from microspheres at 1000 μg/ml and the reference after 24 hours of incubation. MEF cells are seen to cover almost completely the surface and they could proliferate in contact with the extracts from all fabricated microspheres. Furthermore, cells on both samples expressed their typical fibroblastic phenotype and therefore no cytotoxicity effects are detected.

## Conclusions

PHBV microspheres were fabricated with SPIONs^Lauric^ and SPIONs^Oleic^ at different mass ratios through the solid-in-oil-in-water (S/O/W) emulsion-solvent extraction/evaporation method. The method of producing hydrophobic surfaces on magnetic nanoparticles influenced their loading and encapsulation efficiency in the PHBV matrix. The thermal decomposition method induced very low volume of iron nanoparticles coated with oleic acid as compared to the co-precipitation method. This may be due to the narrow particle size distribution of iron nanoparticles. In addition, the DCM used as the oil phase might have caused unstable suspensions especially with SPIONs coated with oleic acid. The chemical structure of oleic acid will be damaged when in contact with organic solvents with higher dielectric constants. This condition resulted in an insufficient encapsulation of SPIONs^Oleic^ in PHBV microspheres. Moreover, it was found that both magnetic susceptibility and iron content are higher in PHBV containing SPIONs^Lauric^. From the cell culture studies, it was revealed that all prepared magnetic PHBV microspheres were biocompatible as they did not lead to any cytotoxicity effect on MEF cells under the experimental conditions investigated. As such, the magnetic microspheres developed in this study represent an attractive technology for biomedical applications.

## Materials and Methods

### Materials

PHBV with a PHV content of 12 wt% was obtained from Goodfellow (Huntingdon, UK). Ringer’s solution and Polyvinyl alcohol (PVA) (MW ~30,000) were bought from Baxter Healthcare (Zürich, Switzerland). Iron (II) chloride tetrahydrate (FeCl_2_⋅4H_2_O), DCM, Propidium iodide (PI) and Triton X-100 were obtained from Sigma-Aldrich (St. Louis, USA). Iron (III) chloride hexahydrate (FeCl_3_⋅6H_2_O) was purchased from Merck (Darmstadt, Germany). Iron reference standards (1 g/L) were bought from Bernd Kraft GmbH (Duisburg, Germany). NH_3_, acetic acid, acetone, sterile Rotilabo® syringe filters with cellulose mixed ester membranes and Spectra/Por 6 dialysis tubing with an MWCO of 10 kDa and a diameter of 29 mm were supplied by Roth (Karlsruhe, Germany).

### Synthesis of superparamagnetic iron oxide nanoparticles (SPIONs)

#### Synthesis of SPIONs^Lauric^ nanoparticles

1.998 g iron (II) and 5.406 g iron (III) salts were dissolved in 20 mL ultrapure water. Under constant stirring and argon flow, 16.5 mL NH_3_ solution (25%) was added at 90 °C to precipitate the iron oxides. To obtain SPIONs^Lauric^, 1.25 g of lauric acid dissolved in acetone was added. The suspension quickly formed a brownish colloid, which was allowed to cool down and was subsequently purified by dialysis. The total iron content was determined using microwave plasma atomic emission spectroscopy (MP-AES). Later the iron oxides were lyophilized and stored at 4 °C until further use.

#### Synthesis of SPIONs^Oleic^ nanoparticles

3.24 g FeCl_3_ .6 H_2_O and 10.95 g sodium oleate were dissolved in a mixture of 24 mL EtOH, 18 mL H_2_O and 42 mL hexane and stirred at room temperature for 5 minutes at 100 rpm and 10 minutes at 250 rpm. The solution was then heated to 70 °C for 4 hours. The organic phase was then separated via a separating funnel and the remaining particles were washed 3 times with water. The ferrofluid was then dried by a lyophilization process. The particles were then dispersed in 61.396 mL 1-octadecan. The particles (10:1) were then washed, once with ethanol, acetone, acetone/ethanol 1:1 and twice with ethanol by centrifugation at 7000 rpm for 20 minutes before drying by lyophilization.

### Preparation of magnetic microspheres

PHBV spherical particles combined with SPIONs^Lauric^ and SPIONs^Oleic^ were produced via solid-in-oil-in-water (S/O/W) emulsion-solvent extraction/evaporation method. In this study, low (6:1) and high (2.5:1) mass ratios of PHBV to SPIONs were investigated. At first, SPIONs^Lauric^ or SPIONs^Oleic^ (S phase) were added into 3 mL of 3% w/v PHBV–DCM solution (O phase). The mixture (S/O) was homogenized by a probe sonicator at 20% power output for 40 seconds. Subsequently, the homogeneous mixture was added with 75 mL of 2% w/v PVA solution (W phase) and emulsified at 10500 rpm for 2 minutes using a homogenizer (T18, IKA, Germany). Later, the obtained emulsion was added into 225 mL of 1% w/v PVA solution. The final solution was stirred at 600 rpm for 2 hours by an over-head stirrer (Eurostar RW 20, IKA, Germany). Then, microspheres were collected by centrifugation (Centrifuge 5430R, Eppendorf, Germany) at 5000 rpm for 4 minutes and washed three times with deionized water before being lyophilized at −50 °C (Alpha 1–2 LDplus, Martin Christ, Germany).

### Characterization

#### FTIR analysis

The chemical state of the magnetic PHBV microspheres was determined using FTIR (IRAffinity-1S, Shimadzu, Japan). Spectra were recorded in absorbance mode at wavenumbers between 4000 and 400 cm^−1^.

#### XRD analysis

The crystalline structure of uncoated SPIONs, SPIONs^Lauric^, SPIONs^Oleic^, PHBV, PHBV incorporated with SPIONs^Lauric^ and SPIONs^Oleic^ was characterized using a Bruker D8 Advance diffractometer (Cu) at room temperature. The peaks were scanned between the 2*Ɵ* angles from 10° to 80° using 0.014°/1 s per step.

#### Thermogravimetric analysis (TGA)

The thermal profiles of pure PHBV microspheres and magnetic PHBV microspheres were determined by TGA (Q5000, TA Instruments, USA). The samples were heated from 27 °C to 550 °C under nitrogen flow. The heating rate was 10 °C/min. The loading and encapsulation efficiencies of SPIONs^Lauric^ and SPIONs^Oleic^ in PHBV were calculated by using eqs (1) and (2), as follows:

1 $$\begin{array}{rcl}{\rm{Loading}}\,{\rm{efficiency}} & = & {\rm{Mass}}\,{\rm{of}}\,{{\rm{SPIONs}}}^{{\rm{Lauric}}{\rm{or}}{\rm{Oleic}}}\,{\rm{in}}\,{\rm{microspheres}}\\  &  & /{\rm{Mass}}\,{\rm{of}}\,{\rm{microspheres}}\times 100 \% \end{array}$$

2 $$\begin{array}{rcl}{\rm{Encapsulation}}\,{\rm{efficiency}} & = & {\rm{Mass}}\,{\rm{of}}\,{{\rm{SPIONs}}}^{{\rm{Lauric}}{\rm{or}}{\rm{Oleic}}}\,{\rm{in}}\,{\rm{microspheres}}\\  &  & /{\rm{Theoretical}}\,{\rm{mass}}\,{\rm{of}}\,{{\rm{SPIONs}}}^{{\rm{Lauric}}{\rm{or}}{\rm{Oleic}}}\times 100 \% \end{array}$$

### Morphology of microspheres

The morphological analysis of the microspheres was performed using scanning electron microscopy (SEM) (Auriga-Zeiss, Germany). The samples for SEM analysis were sputter coated with gold prior to the SEM examination.

### Zeta potential and particle size analysis

The zeta potential and particle size of microspheres were analyzed by Malvern Zetasizer Nano ZS and Mastersizer particle size analyzer 2000 (Malvern, Worcestershire, UK), respectively. The measurements were carried out by suspending the microspheres in deionized water. The mean particle size of the microspheres was determined based on the peak mean of number size distribution approach.

### Magnetic susceptibility and iron content measurement

Magnetic properties of the microspheres were determined by magnetic susceptibility measurements (MS2G, Bartington, Witney, Oxfordshire, UK). At first, 1 mg of dried microspheres was placed in the measuring tube. Then the tube was positioned in the column or aperture with the magnetic susceptibility sensors and systems in order to obtain the data. For the iron content determination, the microspheres were dispensed in 65% HNO_3_ and diluted with deionized water (1:9) prior to the detection of iron in the microspheres via microwave plasma atomic emission spectroscopy (MP-AES, Agilent 4200).

### Cytocompatibility assays

PHBV microspheres, PHBV incorporated with SPIONs^Lauric^ and SPIONs^Oleic^ were prepared with cell culture medium (DMEM) with 2% antibiotic at concentrations of 1 μg/ml, 10 μg/ml, 100 μg/ml and 1000 μg/ml and later incubated for 12 hours. Mouse embryotic fibroblast (MEF) cells were used for cytotoxicity test according to ISO standard 10993–5. In detail, MEF cells were grown in cell culture flasks containing DMEM, 10% FCS, 1% non-essential amino acids and 1% antibiotic (penicillin and streptomycin) for 48 hours. The cells were harvested by trypsin and 50,000 cells were seeded on each well and also incubated for 12 hours. Then, 0.5 ml of the release product (extract) from the microspheres at different concentrations was placed in contact with the cells and incubated for 24 hours. All incubation processes were carried out in a humidified atmosphere of 95% relative humidity and 7.5% CO_2_, at 37.8 °C.

The vitality of MEF cells grown on the supernatant at different concentrations of microspheres was assessed through the enzymatic conversion of tetrazolium salt (WST-8 assay kit, Sigma Aldrich, Germany) after 24 hours of cultivation. The release product of microspheres was completely removed from the cells and 0.3 ml of freshly prepared culture medium was added containing 1 vol % WST-8 solution, followed by incubation for 2 hours. Subsequently, 100 μL of supernatant from each sample was transferred into a 96 well-plate and the absorbance was measured at 450 nm with a microplate reader (PHOmo, Autobio Labtec Instruments Co. Ltd, China). To assess the viability and morphology of cells, Hematoxylin and Eosin (H&E) stains were introduced to the cells. Hematoxylin and Eosin have a deep blue-purple and pink colour which stains nucleic acids and cytoplasm of the cells, respectively. The images of stained cells were taken by using a microscope (AxioCam ERc 5s, Primovert, Carl Zeiss, GmbH, Germany).

### Statistics

Statistical analysis of viability of MEF cells was conducted by one-way analysis of variance (ANOVA) on PHBV, PHBV/SPIONs^Lauric^ and PHBV/SPIONs^Oleic^ microspheres after 24 hours of incubation. The pairwise comparison of the means was performed with the Bonferroni’s test (post hoc comparison). A value of *P* < 0.05 was considered statistically significant.
